# Characterization of the ultra-short echo time magnetic resonance (UTE MR) collagen signal associated with myocardial fibrosis

**DOI:** 10.1186/1532-429X-17-S1-Q7

**Published:** 2015-02-03

**Authors:** Adrienne G Siu, Andrew Ramadeen, Xudong Hu, Lily Morikawa, Li Zhang, Justin Lau, Garry Liu, Mihaela Pop, Kim A Connelly, Paul Dorian, Graham A Wright

**Affiliations:** 1 Department of Medical Biophysics, University of Toronto, Toronto, ON, Canada; 2 Imaging Research, Sunnybrook Research Institute, Toronto, ON, Canada; 3 Keenan Research Centre, Li Ka Shing Knowledge Institute, Toronto, ON, Canada; 4 Centre for Modeling Human Disease, Toronto Centre for Phenogenomics, Toronto, ON, Canada; 5 Division of Cardiology, St. Michael's Hospital, Toronto, ON, Canada

## Background

The homogeneous distribution of collagen in diffuse myocardial fibrosis renders the disease unsuitable for imaging using late gadolinium enhancement (LGE) [1]. More recently, the estimation of extracellular volume from T_1_ maps involving gadolinium agents has shown promise; however, these methods are not specific to collagen and are governed by gadolinium kinetics [2]. The diagnosis of diffuse myocardial fibrosis would benefit from an imaging method that can directly detect collagen. Notably, ultra-short echo time magnetic resonance (UTE MR) is a technique that can be used to detect short T_2_* species, including collagen [3]. Our objective is to characterize the UTE signal of protons in the collagen molecule, including their T_2_* and chemical shift. Direct isolation of a collagen signal could aid in the diagnosis of myocardial fibrosis, especially for diffuse distributions, and the assessment of disease extent.

## Methods

Collagen solutions of concentrations ranging from 0 % m/v to 50 % m/v were prepared by dissolving hydrolyzed type I and III collagen powder in 0.125 mM MnCl_2_ , where the signal decay of MnCl_2_ mimicked that of cardiac muscle. Each solution was scanned using a 3D UTE pulse sequence at 7 T, acquiring TEs from 0.02 ms to 25 ms, at a resolution of 0.781 mm isotropic. Upon fitting with a model of bi-exponential T_2_* with oscillation, the UTE collagen signal fraction was determined and calibrated against the collagen concentration. The T_2_* and resonance frequency (arising from the chemical shift) of collagen were assessed in collagen solutions. Validation of the collagen signal properties was also performed in formalin-fixed canine heart tissue, imaged with TEs from 0.02 ms to 25 ms, at a resolution of 0.156 mm isotropic.

## Results

**Figure 1:**
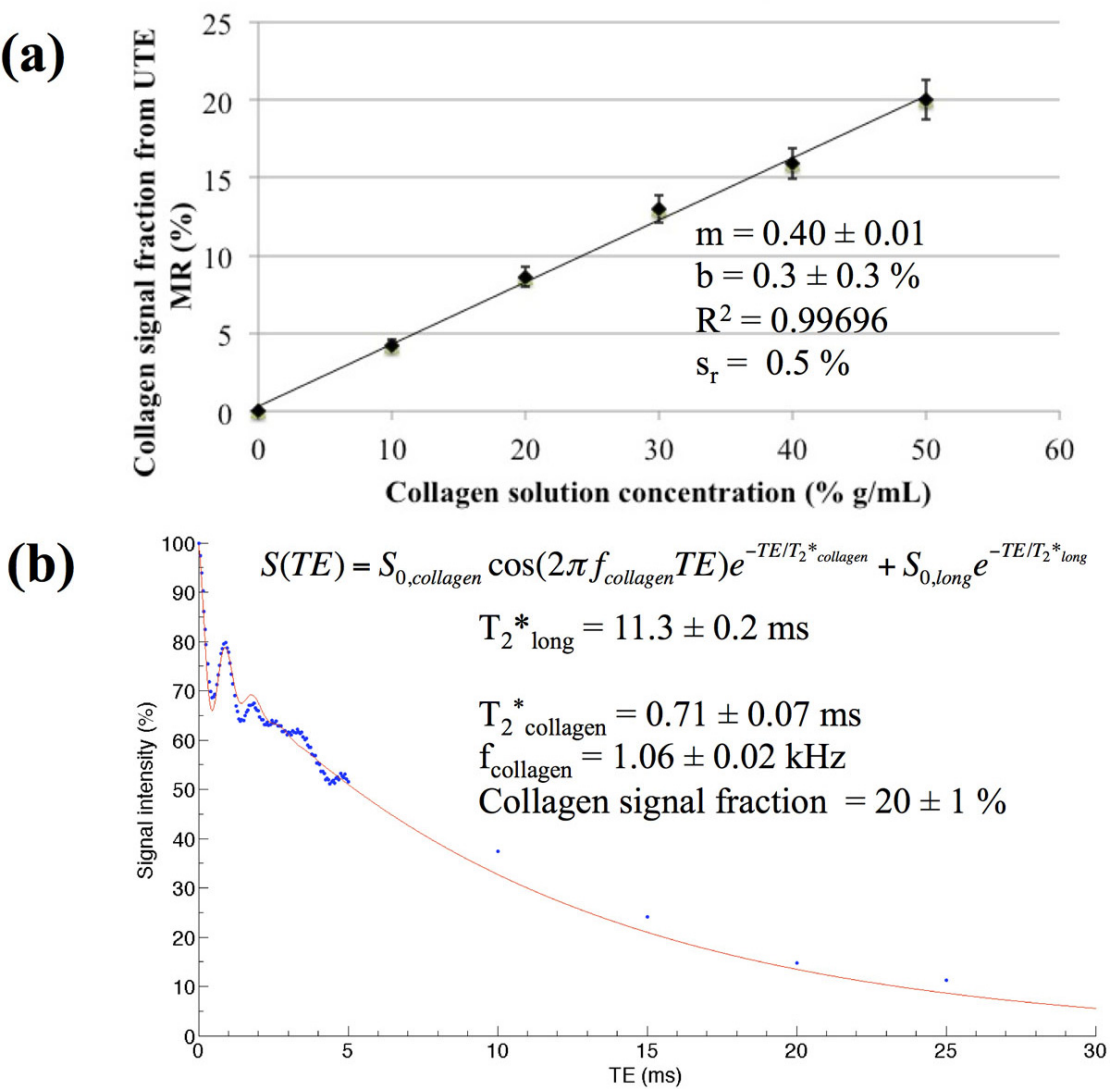
UTE results in collagen solutions. (a) Collagen solution calibration plot, demonstrating a linear relationship between the UTE collagen signal fraction and the collagen concentration. m = slope, b = y-intercept, R^2^ = correlation coefficient, s_r_ = standard deviation about the regression. (b) T_2_* decay of the 50 % collagen solution, fitted using a bi-exponential T_2_* model with oscillation. T_2_*_long_ denotes the long T_2_* of MnCl_2_ (mimicking cardiac muscle). Although not all long TEs were fitted, the focus was in the characterization of the short TEs ≤ 2 ms, where the T_2_* model is accurate.

**Figure 2:**
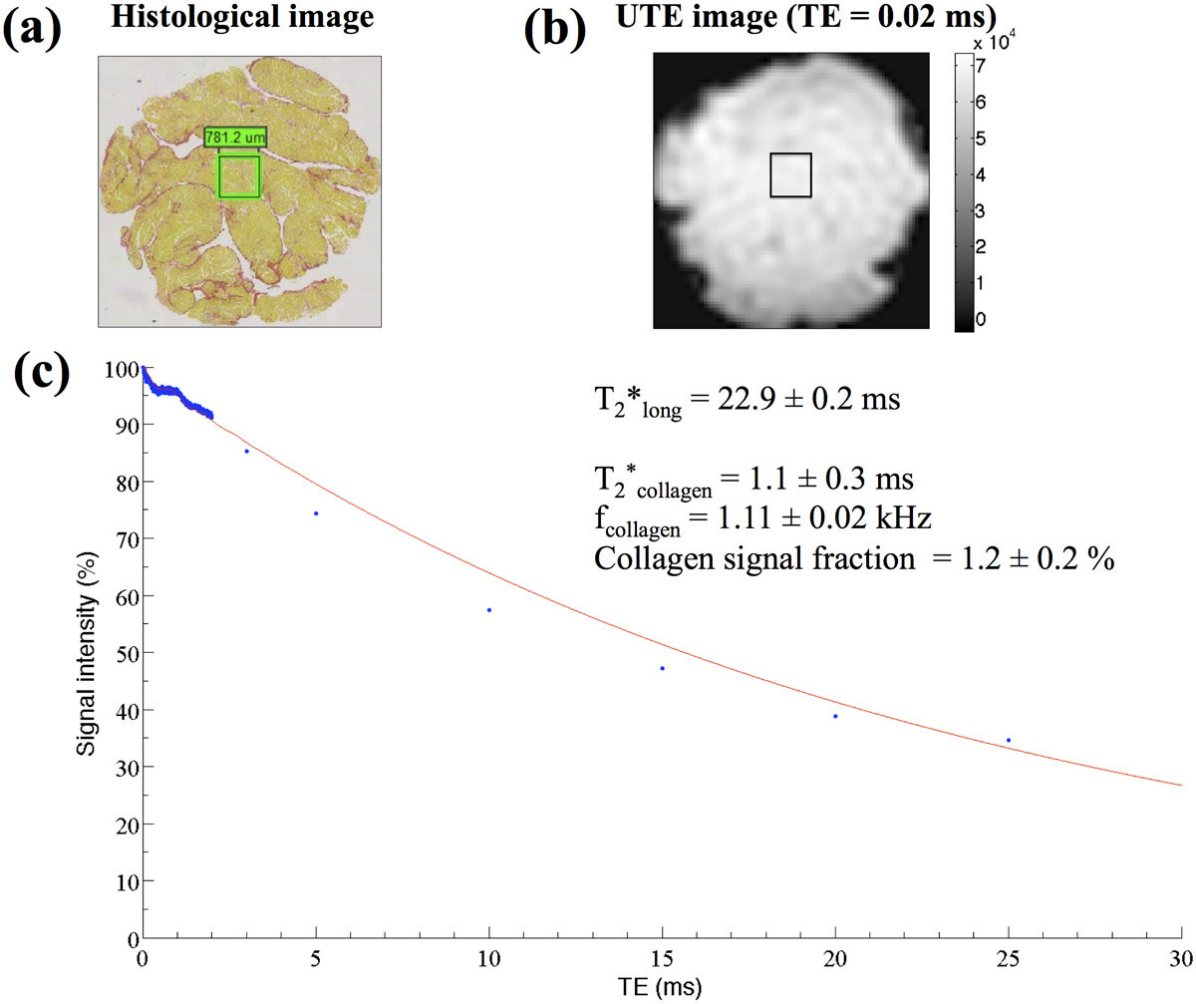
Histology and UTE results in canine heart tissue. (a) Histological slice of heart tissue, stained with Picrosirius Red. The 781.2 μm x 781.2 μm region-of-interest (ROI) used for analysis is delineated. The collagen area fraction in the ROI was determined to be 4 ± 2 %, based on a pixel threshold algorithm. (b) Corresponding UTE MR image at TE = 0.02 ms, with the ROI delineated. (c) T_2_* decay within the ROI. T_2_*_long_ denotes the long T_2_* of cardiac muscle. TEs ≤ 2 ms were finely sampled to determine the collagen T_2_* and resonance frequency, where the T_2_* model is accurate. Based on the calibration plot in Figure 1a, the collagen signal fraction of 1.2 ± 0.2 % was equivalent to a collagen concentration of 2.3 ± 0.9 %. Hence, there was agreement between the collagen area fraction determined from histology (4 ± 2 %) and the collagen concentration.

For collagen concentrations of 10 % to 50 %, the mean collagen T_2_* was 0.75 ± 0.05 ms, and the mean collagen frequency was 1.061 ± 0.004 kHz. A linear relationship (slope = 0.40 ± 0.01, R^2^ = 0.99696) was determined between the UTE collagen signal fraction associated with these characteristics and the measured collagen concentration (Figure 1). Similarly in canine heart tissue, a signal with T_2_* of 1.1 ± 0.3 ms and resonance frequency of 1.11 ± 0.02 kHz upfield of water was determined, consistent with collagen (Figure 2). The UTE collagen signal fraction of 1.2 ± 0.2 % in tissue corresponded to a collagen concentration of 2.3 ± 0.9 %, which was within the uncertainty of the collagen area fraction determined from histology (4 ± 2 %).

## Conclusions

The results suggest that collagen associated with myocardial fibrosis can be endogenously detected and quantified using UTE MRI. This signal is specific to protons in collagen, characterized by a T_2_* of ~ 0.8 ms and a resonance frequency of ~ 1.1 kHz upfield of water at 7 T. Such properties would be beneficial in the determination of collagen content due to disease.

## Funding

Canadian Institutes of Health Research (CIHR).
